# *CORR* Insights^®^: Standard Comorbidity Measures Do Not Predict Patient-reported Outcomes 1 Year After Total Hip Arthroplasty

**DOI:** 10.1007/s11999-015-4252-7

**Published:** 2015-03-20

**Authors:** J. Christiaan Keurentjes

**Affiliations:** Department of Orthopaedic Surgery, Leiden University Medical Center, Albinusdreef 2, 2333 ZA Leiden, The Netherlands

## Where Are We Now?

A majority of patients who undergo hip replacement surgery report good clinical outcomes at 1 year followup. This is reflected in high-mean utility scores (EQ-5D index), high-mean VAS scores of self-reported health state (EQ-VAS), patient satisfaction scores with the outcomes of surgery (VAS Satisfaction), and low-mean VAS scores of pain (VAS Pain) [4]. However, approximately 9% of patients report persisting pain after surgery [2], and approximately 16% are not completely satisfied with their surgical results [3]. This large variance—a majority of patients with good patient reported outcome measure (PROM) scores, but a meaningful minority with poor PROM scores—can partially be explained by patient age, gender, preoperative health status, or comorbidities.

The current study by Greene and colleagues combines the detail of a multicenter cohort study nested within the solid framework provided by a national joint registry and serves as an example for future PROM studies [4]. Greene and colleagues have shown that detailed comorbidity measures have no added value to the preoperative Charnley classification in explaining PROM score variability [4]. These important findings simplify future PROM research: In order to account for patient comorbidities, we only need to know whether (1) the other hip is affected, and (2) whether the patient suffers from other joint pain or has any comorbidity which affects her/his ability to ambulate.

## Where Do We Need To Go?

Because therapeutic options for patients with poor outcomes after total hip replacement are limited, it is important to try to identify those patients at highest risk for complications or dissatisfaction before they undergo the procedure. Low-risk patients could undergo hip replacement immediately and, ideally, high-risk patients would undergo perioperative optimization in order to lower the risk of a poor outcome. The perfect prediction model would allow for an accurate prediction of the risk of a poor outcome, based on a number of readily available predictors, and it would explain close to 100% of the variance of the PROM score. Unfortunately, no such model exists now.

Greene and colleagues have shown that roughly 10% of the variance in the EQ-5D index and EQ-VAS scores can be explained by the Charnley classification and the preoperative PROM score [4]. Three percent of the VAS Pain could be explained by the Charnley classification and the preoperative PROM score and 1% of the VAS Satisfaction could be explained by the Charnley classification [4]. These r^2^-values probably underestimate the true explained PROM score variance because the PROM scores are not normally distributed but left-skewed. However, it is clear that there is much room for improvement in all four studied PROMs, and in the predictive models that we can derive from them.

## How Do We Get There?

Much of the PROM score variance described in the study by Greene and colleagues is currently unexplained. The added value of new predictors should be studied in conjunction with all currently known predictors. Candidate predictors include the preoperative radiographic severity of osteoarthritis, which appears relevant in some PROM dimensions [8]. The role of other patient characteristics, such as the highest attained level of education, is more controversial [1, 5, 6].

Future studies should not only focus on discovering unknown predictors, but should also try to replicate findings of previous studies, thereby minimizing the risk of publication bias. Both discovery and replication studies need consecutive cohorts of hip replacement patients. Future studies should also use the probability of a clinically important difference or patient acceptable symptom state as a primary outcome measure, as these probabilities are more relevant for individual patients we encounter in clinical practice who either do or do not achieve an acceptable state or relevant improvement [11]. Recently, minimal clinically important differences and patient acceptable symptom states have been estimated for the SF-36 [7], EQ-5D, HOOS [10], and Oxford Hip Score [9]. Those findings offer helpful thresholds that can help us construct more-robust predictive models from data available in national registries using those endpoints, and, perhaps, to identify those patients at greatest risk for persistent pain or disability after hip replacement.

## References

[1] Allen BR, Rosenzweig S, Myers L, Barrack RL (2011). The Frank Stinchfield Award: The impact of socioeconomic factors on outcome after THA: A prospective, randomized study. Clin Orthop Relat Res..

[2] Beswick AD, Wylde V, Gooberman-Hill R, Blom A, Dieppe P (2012). What proportion of patients report long-term pain after total hip or knee replacement for osteoarthritis? A systematic review of prospective studies in unselected patients. BMJ Open..

[3] Espehaug B, Havelin LI, Engesaeter LB, Langeland N, Vollset SE (1998). Patient satisfaction and function after primary and revision total hip replacement. Clin Orthop Relat Res..

[4] Greene ME, Rolfson O, Nemes S, Gordon M, Malchau H, Garellick G (2014). Education attainment is associated with patient-reported outcomes: Findings from the Swedish Hip Arthroplasty Register. Clin Orthop Relat Res..

[5] Greene ME, Rolfson O, Gordon M, Garellick G, Nemes S. Standard comorbidity measures do not predict patient-reported outcomes 1 year after total hip arthroplasty. [Published online ahead of print February 21, 2015]. *Clin Orthop Relat Res*. DOI: 10.1007/s11999-015-4195-z. 10.1007/s11999-015-4195-z PMC4586242 25700999

[6] Keurentjes JC, Blane D, Bartley M, Keurentjes JJ, Fiocco M, Nelissen RG. Socio-economic position has no effect on improvement in health-related quality of life and patient satisfaction in total hip and knee replacement: A cohort study. *PloS One*. 2013: e56785. 10.1371/journal.pone.0056785 PMC3592876 23520456

[7] Keurentjes CJ, Fiocco M, Nelissen RG (2014). Willingness to undergo surgery again validated clinically important differences in health-related quality of life after total hip replacement or total knee replacement surgery. J Clin Epidemiol..

[8] Keurentjes JC, Fiocco M, So-Osman C, Onstenk R, Koopman-Van Gemert AW, Pöll RG, Kroon HM, Vliet Vlieland TP, Nelissen RG (2013). Patients with severe radiographic osteoarthritis have a better prognosis in physical functioning after hip and knee replacement: A cohort-study. PLoS One.

[9] Keurentjes JC, Van Tol FR, Fiocco M, So-Osman C, Onstenk R, Koopman-Van Gemert AW, Pöll RG, Nelissen RG. Patient acceptable symptom states after total hip or knee replacement at mid-term follow-up: Thresholds of the Oxford hip and knee scores. *Bone Joint Res.* 2014;3:7–13. 10.1302/2046-3758.31.2000141 PMC3928564 24421318

[10] Paulsen A, Roos EM, Pedersen AB, Overgaard S (2014). Minimal clinically important improvement (MCII) and patient-acceptable symptom state (PASS) in total hip arthroplasty (THA) patients 1 year postoperatively: A prospective cohort study of 1,335 patients. Acta Orthop.

[11] Singh J, Sloan JA, Johanson NA (2010). Challenges with health-related quality of life assessment in arthroplasty patients: Problems and solutions. J Am Acad Orthop Surg.

